# Perioperative comparison of whether the liver was lifted out of the abdominal cavity in Kasai surgery

**DOI:** 10.3389/fped.2025.1549636

**Published:** 2025-02-20

**Authors:** Ke Xu, Juan Gao, Xiaoyi Chen, Yifan Fang, Dianming Wu, Yu Lin

**Affiliations:** ^1^ Department of General Surgery, Fujian Children's Hospital (Fujian Branch of Shanghai Children's Medical Center), College of Clinical Medicine for Obstetrics & Gynecology and Pediatrics, Fujian Medical University, Fuzhou, China; ^2^ Department of Child Health Care, Fujian Provincial Maternity and Children's Hospital, Fuzhou, China; ^3^ Department of Hospital Infection Management, Fuzhou Jinan District Hospital, Fuzhou, China

**Keywords:** biliary atresia, Kasai surgery, liver, perioperative period, comparison

## Abstract

**Objective:**

To compare the perioperative effect of whether the liver is lifted out of the abdominal cavity during Kasai surgery in patients with biliary atresia type III.

**Methods:**

We retrospectively analyzed 29 males and 33 females aged 2.2 ± 0.6 months who underwent kasai surgery from June 2019 to December 2022 at Fujian Provincial Children's Hospital. Among the 62 children with the liver not lifted out of the abdominal cavity into the experimental group (*n* = 31), and those with the liver lifted out of the abdominal cavity into the control group (*n* = 31). We compared the operation time, surgical incision length, intraoperative blood pressure and body temperature fluctuations, average daily peritoneal drainage, postoperative length of hospital stay, postoperative complications, postoperative total bilirubin level, and autologous liver survival rate between the two groups.

**Results:**

The length of the surgical incision in the control group (7.5 ± 1.2 cm) was longer than that in the experimental group (6.7 ± 1.1 cm), and the difference was statistically significant (t = 2.88, *P* = 0.005). The fluctuations in blood pressure in the control group (before and after the liver was lifted out of the abdominal cavity) (24.3 ± 7.7 mmHg) were greater than that in the experimental group (before and after hooking to expose the hilar area) (20.2 ± 6.0 mmHg), which was a statistically significant difference (t = 2.32, *P* = 0.023). The temperature fluctuations in the control group (before and after the liver was lifted out of the abdominal cavity) (0.3 ± 0.1°C) was greater than that in the experimental group (before and after the liver was lifted out of the abdominal cavity) (0.1 ± 0.1°C), showing a statistically significant difference (t = 8.19, *P* = 0.000). The average daily abdominal drainage in the control group was 71.3 ± 33.5 ml, which was greater than that in the experimental group (49.2 ± 49.6 ml), and the difference was statistically significant (t = 2.06, *P* = 0.044). The number of days of postoperative hospital stay in the control group (18.2 ± 4.8 days) was significantly more than that in the experimental group (15.8 ± 3.8 days), with a statistically significant difference (t = 2.18, *P* = 0.033).

**Conclusion:**

In children with biliary atresia type III, the liver is not lifted out of the abdominal cavity during Kasai surgery increases the safety of surgery and reduces the length of hospital stay.

## 1 Introduction

Biliary atresia (BA) is an occlusive lesion affecting the intrahepatic and external bile ducts, leading to cholestasis and progressive liver fibrosis until liver cirrhosis, which is life-threatening in children (1). The incidence of BA is about 1:5,000–12,000, with a higher incidence in Asia (2). The classification of BA depended on the different parts of the atresia of the extrahepatic bile duct, namely: type I: common bile duct atresia (5%); Type II: hepatic duct atresia (3%); Type III: hilar atresia (92%) (3). Type III BA is the most common, and postoperative jaundice clearance and autologous hepatic survival rates are worse than those of type I and II (4). Surgical interventions are the primary treatment for BA, consisting of portoenterotomy (Kasai procedure), laparoscopic kasai surgery, and liver transplantation (5). The Kasai surgery is currently the preferred procedure for the treatment of type III biliary atresia, and the key to this procedure is to isolate the hepatic fibrotic mass and dissect the blood vessels (6–8). In the traditional Kasai surgery, to better expose and anatomize the hilar area, the surgeon often adopts a large transverse incision in the upper abdomen to free the ligaments around the liver and then elevate it out of the abdominal cavity, which has reached a consensus at the European Biliary Atresia Conference (9). However, the removal of the liver from the abdominal cavity during the Kasai surgery often brings many adverse effects to the perioperative period of the child, such as instability of intraoperative blood pressure, body temperature, and even interruption of the operation. It has been demonstrated that lifting the liver out of the abdominal cavity during Kasai surgery has resulted in decreasing in blood pressure in 50 percent of children (10). Therefore, in the subsequent Kasai surgery, the author's unit tried not to lift the liver out of the abdominal cavity during the operation, and achieved good results. This study will compare and explore the effect of whether or not to remove the liver from the abdominal cavity during Kasai surgery on the perioperative period of children.

## 2 Methods

### 2.1 Clinical data

We retrospectively analyzed the data of children with biliary atresia type III who received Kasai surgery from August 2020 to August 2024 at Fujian Provincial Children's Hospital. A total of 62 cases were enrolled, including 29 males and 33 females, aged 2.2 ± 0.6 months. Among the 62 children, those without liver removal from the abdominal cavity were in the experimental group (31), and those with liver removal from the abdominal cavity were in the control group (31). There were no significant differences in age, sex ratio, and weight between the two groups (*P* > 0.05). All surgeries were performed by the same same surgeons.Inclusion criteria: (1) Children with type III biliary atresia;(2) patients who successfully underwent kasai surgery; (3) complete clinical data. Children with other liver diseases, such as congenital choledochal cysts, viral hepatitis, and Alagille syndrome, were excluded.

### 2.2 Operative technique

**Figure 1:**
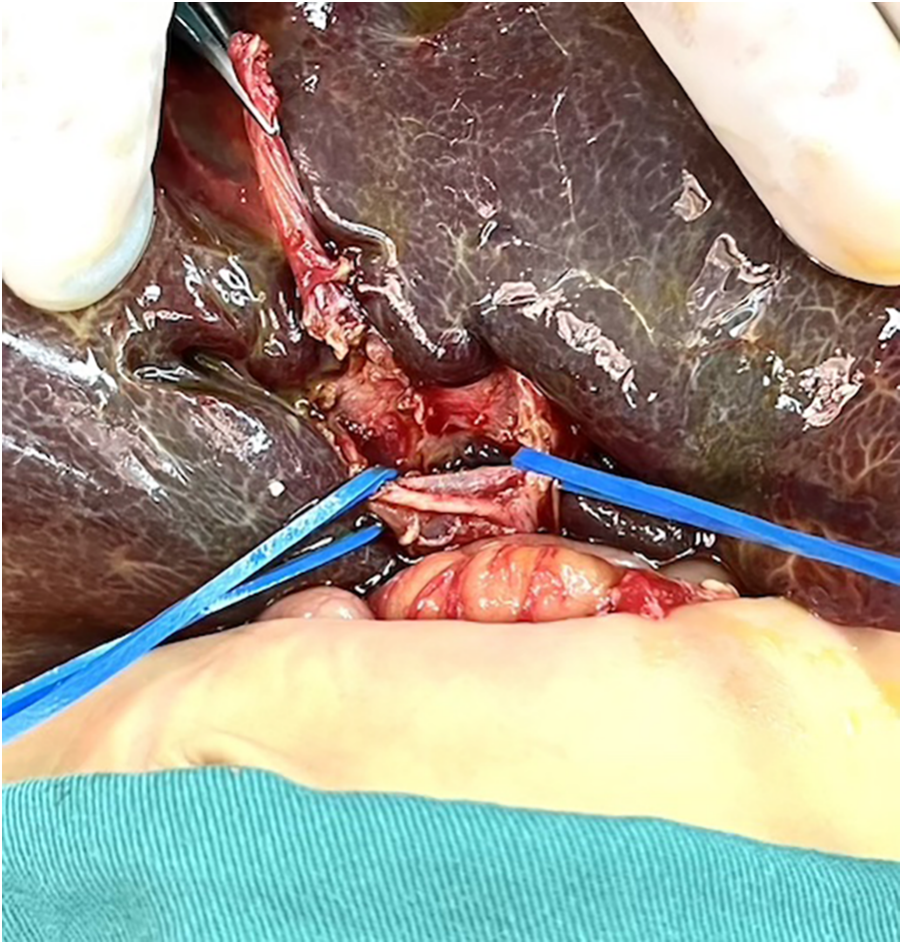
The liver was lifted out of the abdominal cavity.

**Figure 2:**
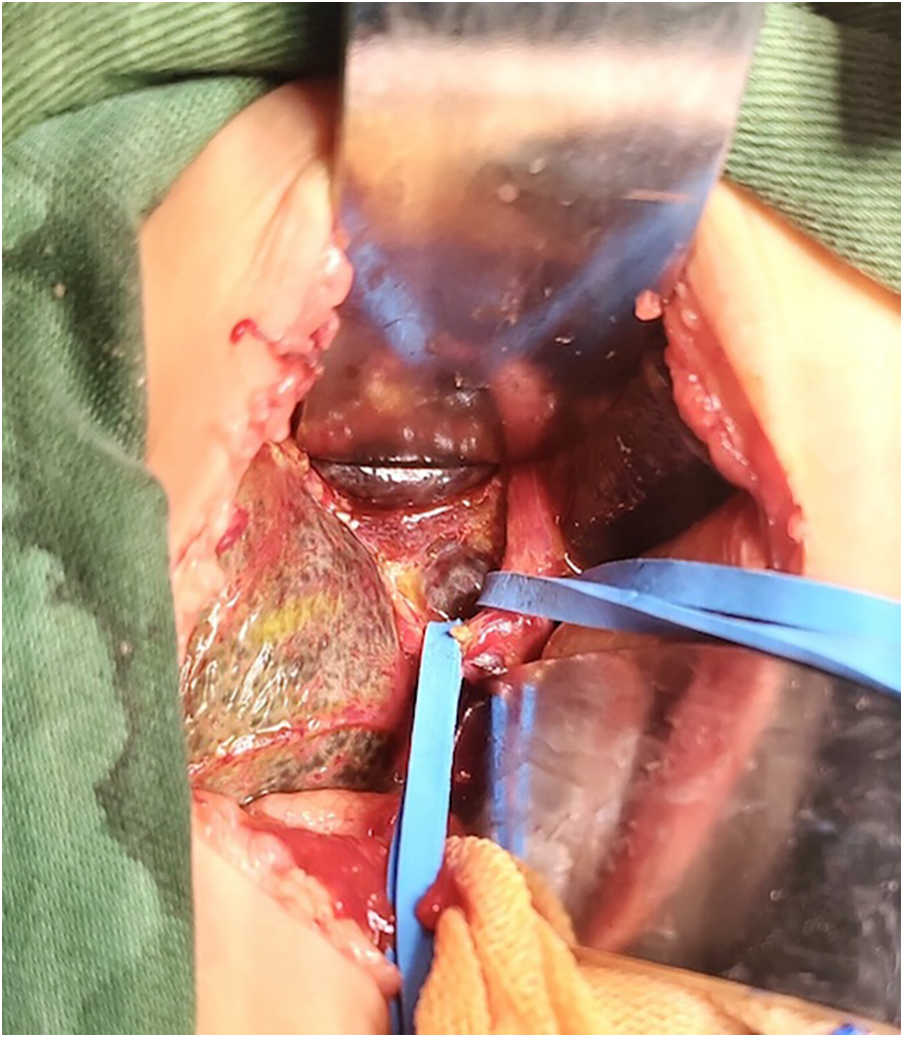
The liver was not lifted out of the abdominal cavity.

(1) The control group took an arc-shaped incision of about 8.0 cm under the right costal margin, incising the abdominal wall layer by layer into the abdominal cavity. After dissociating the perihepatic ligament, a large portion of the liver is lifted out of the incision and turned out the facies inferior hepatis (Figure 1). The fibrous cord-like gallbladder and extrahepatic bile duct along the hilum of the liver show a hilar fibrous mass and pull the fibrotic extrahepatic biliary duct to expose the visual field. Completely freeing the left and right branches of the portal vein and the left and right branches of the hepatic artery in the hepatic hilar area, stretching with elastic traction to expose the hepatic hilar fibrous masses, and transecting the fibrous masses on the surface of the liver parenchym. Gauze compresses the section to stop bleeding, and care is taken not to use electrocoagulation to stop bleeding as much as possible. Cut the jejunum 15.0 cm from the ligament of Treitz, suture the distal jejunum, and then elevate the porta hepatis through the posterior part of the transverse colon. The intestinal wall of the contralateral margin of the mesangium was cut about 1.5 cm and partially anastomosed side by side with the fibrous mass of the porta hepatis. Complete the Y-shaped anastomosis between the jejunum and the proximal jejunum at 30.0 cm from the hepato-enteric anastomosis. A silicone drain is placed below the hepatic hilar anastomosis. (2) In the experimental group, the subcostal arc incision was only about 6 cm. Without requiring complete freeing of the perihepatic ligament and lifting the liver out of the abdominal cavity during the operation, only by exposing the hepatic hilum with a retractor (Figure 2). The remaining operations were the same as those in the control group. For some patients exposing porta hepatis difficulty, the square lobe of the liver can be suspended anterior to the hepatic hilum by suturing or even partially resected to expose the visual field, with the resected portion available for liver biopsy.

### 2.3 Observation indicators and follow-up

Age, gender, weight, operation time, surgical incision length, intraoperative blood pressure and body temperature fluctuations, daily intraperitoneal drainage, number of postoperative hospitalizations, postoperative total bilirubin level, the number of cases with complete jaundice resolution 3 months after surgery, postoperative complications (including frequent cholangitis and biliary fistula), and autologous liver survival rate (no liver transplant survivors after Kasai surgery). This study mainly followed up on postoperative liver function, cholangitis, and liver transplantation through outpatient reexamination, phone calls, and WeChat messages. The follow-up was 12–66 months.

### 2.4 Statistical processing

Independent samples *t*-test was used for the comparison of continuous data, and the ×^2^ test was employed for the comparison of count data. *P* < 0.05 was statistically significant. We utilized SPSS 26.0 software for all data processing.

## 3 Result

### 3.1 General information

There was no significant difference in age, gender, and weight between the two groups (*P* > 0.05) (Table 1).

**Table 1:** Comparison of the general data and intraoperative conditions of the two groups.

Variables	Control group (*n* = 31)	Experimental group (*n* = 31)	*t*/*χ*^2^	*P*
Male [*n* (%)]	14 (45.2)	15 (48.4)	0.065	0.799
Age (m, $\bar{x}\pm s$)	2.2 ± 0.6	2.3 ± 0.7	−0.42	0.678
Weight (kg, $\bar{x}\pm s$)	5.0 ± 0.6	4.8 ± 0.6	1.40	0.623
Operation time (h, $\bar{x}\pm s$)	3.5 ± 0.8	3.4 ± 0.6	0.64	0.524
Daily abdominal drainage (ml, $\bar{x}\pm s$)	71.3 ± 33.5	49.2 ± 49.6	2.06	0.044
Length of the surgical incision (cm, $\bar{x}\pm s$)	7.5 ± 1.2	6.7 ± 1.1	2.88	0.005
Fluctuations of blood pressure (mmhg, $\bar{x}\pm s$)	24.3 ± 7.7	20.2 ± 6.0	2.32	0.023
Fluctuations of body temperature (℃, $\bar{x}\pm s$)	0.3 ± 0.1	0.1 ± 0.1	8.19	0.000

### 3.2 Intraoperative comparison

There was no significant difference in operation time between the two groups (*P* > 0.05). The length of the surgical incision in the control group (7.5 ± 1.2 cm) was greater than that in the experimental group (6.7 ± 1.1 cm), and the difference was statistically significant (t = 2.88, *P* = 0.005). The fluctuations in blood pressure in the control group (before and after the liver elevated out of the abdominal cavity) (24.3 ± 7.7 mmHg) was greater than that in the experimental group (before and after the hook exposed the hilar area) (20.2 ± 6.0 mmHg), representing a statistically significant difference (t = 2.32, *P* = 0.023). The body temperature fluctuations in the control group (before and after the liver was lifted out of the abdominal cavity) (0.3 ± 0.1°C) was more than that in the experimental group (before and after the liver was lifted out of the abdominal cavity) (0.1 ± 0.1°C), with a statistically significant difference (t = 8.19, *P* = 0.000). The average daily abdominal drainage in the control group was (71.3 ± 33.5 ml) was greater than that in the test group (49.2 ± 49.6 ml), which was a statistically significant difference (t = 2.06, *P* = 0.044).

### 3.3 Comparison of postoperative conditions

The number of postoperative hospitalization days in the control group (18.2 ± 4.8) days was greater than that in the experimental group (15.8 ± 3.8) days, indicating a statistically significant difference (t = 2.18, *P* = 0.033). The number of children with total bilirubin preoperatively, total bilirubin at 3 months after surgery, and complete jaundice resolution 3 months after surgery did not differ significantly between the control group and the experimental group, respectively, 127.8 ± 35.7 umol/L vs. 124.1 ± 39.5 umol/L, 31.1 ± 31.7 umol/L vs. 38.3 ± 42.9 umol/L, and 15 cases (48.4%) vs. 16 cases (51.6%) (*P* > 0.05). The number of AST, ALT, γ-GGT, ALP before operation and AST, ALT, γ-GGT, ALP 2 weeks after operation did not differ significantly between the control group and experimental group, respectively, 218.8 ± 147.6 U/L vs. 201.5 ± 120.6 U/L, 217.1 ± 192.4 U/L vs. 209.3 ± 190.1 U/L, 519.5 ± 284.3 U/L vs. 501.3 ± 410.1 U/L, 523.9 ± 184.3 U/L vs. 578.5 ± 227.7 U/L, 202.8 ± 111.8 U/L vs. 205.5 ± 116.2 U/L, 236.4 ± 177.8 U/L vs. 224.2 ± 146.3 U/L, 745.4 ± 495.5 U/L vs. 621.4 ± 481.6 U/L, 377.8 ± 126.1 U/L vs. 396.9 ± 156.4 U/L (*P* > 0.05).The control group had 22 cases (71.0%) with polychondritis and no biliary fistula. The experimental group had 19 cases (61.3%) with polychondritis and 1 case (3.2%) with biliary fistula. The incidence of postoperative polychondritis and biliary fistula was not significantly different in both groups (*P* > 0.05) (Table 2).

**Table 2:** Comparison of postoperative indexes between the two groups.

Variables	Control group (*n* = 31)	Experimental group(*n* = 31)	*t*/*χ*^2^	*P*
Postoperative hospital days (d, $\bar{x}\pm s$)	18.2 ± 4.8	15.8 ± 3.8	2.18	0.033
Total bilirubin preoperatively (umol/L, $\bar{x}\pm s$)	127.8 ± 35.7	124.1 ± 39.5	0.39	0.697
Total bilirubin at 3 months after surgery (umol/L, $\bar{x}\pm s$)	31.1 ± 31.7	38.3 ± 42.9	−0.75	0.456
Complete jaundice resolution 3 months after surgery [*n* (%)]	15 (48.4)	16 (51.6)	0.07	0.799
Frequent cholangitis [*n* (%)]	22 (71.0)	19 (61.3)	0.65	0.422
Biliary fistula [*n* (%)]	0 (0)	1 (3.2)	1.02	0.313
Preoperative AST(U/L)	218.8 ± 147.6	201.5 ± 120.6	0.51	0.615
Preoperative ALT(U/L)	217.1 ± 192.4	209.3 ± 190.1	0.16	0.874
Preoperative γ-GGT(U/L)	519.5 ± 284.3	501.3 ± 410.1	0.12	0.908
Preoperative ALP(U/L)	523.9 ± 184.3	578.5 ± 227.7	−1.04	0.303
Postoperative AST(U/L)	202.8 ± 111.8	205.5 ± 116.2	−0.90	0.928
Postoperative ALT(U/L)	236.4 ± 177.8	224.2 ± 146.3	0.30	0.769
Postoperative γ-GGT(U/L)	745.4 ± 495.5	621.4 ± 481.6	1.00	0.322
Postoperative ALP(U/L)	377.8 ± 126.1	396.9 ± 156.4	−0.53	0.597

### 3.4 Autologous liver survival rate

21 patients (67.7%) in the control group did not undergo liver transplantation 1 year after surgery, and all of them survived. In the experimental group, 23 patients (74.2%) survived without liver transplantation at 1 year after surgery. In the control group, 18 patients (58.1%) had no liver transplantation at 2 years postoperatively, and all survived. In the experimental group, 20 patients (64.5%) did not proceed to liver transplantation 2 years after surgery, and all of them survived.

## 4 Discussion

BA is one of the leading causes of neonatal jaundice and is a severe life-threatening disease. In terms of treatment, the Kasai surgery is the first-line surgical treatment for biliary atresia. The continuous improvement of Kasai surgery by scholars at home and abroad has resulted in a postoperative bile excretion rate of up to 70%, which saves the lives of many children and delaying the need for liver transplantation (11). In traditional Kasai surgery, to expose the porta hepatis and dissect the fibrous masses in the porta hepatis better, the liver is usually lifted out of the abdominal cavity by making a big transverse incision in the upper abdomen, which has also been the case for a long time in the author's unit. This study improved on the above procedure by no longer completely freeing the perihepatic ligaments and lifting the liver out of the abdominal cavity during the Kasai procedure but instead using retractors to expose and suture, lifting a portion of the hepatic lobe anterior to the hilar portal, and resecting a portion of the hepatic lobe if necessary, This improvement has yielded excellent results, as evidenced by a much lower risk of surgical anesthesia, a more aesthetic surgical wound, a reduction in postoperative abdominal drainage, a shorter hospital stay, no prolongation of surgical time, no compromise of surgical outcome, and no increase in surgical complications.

**Figure 3:**
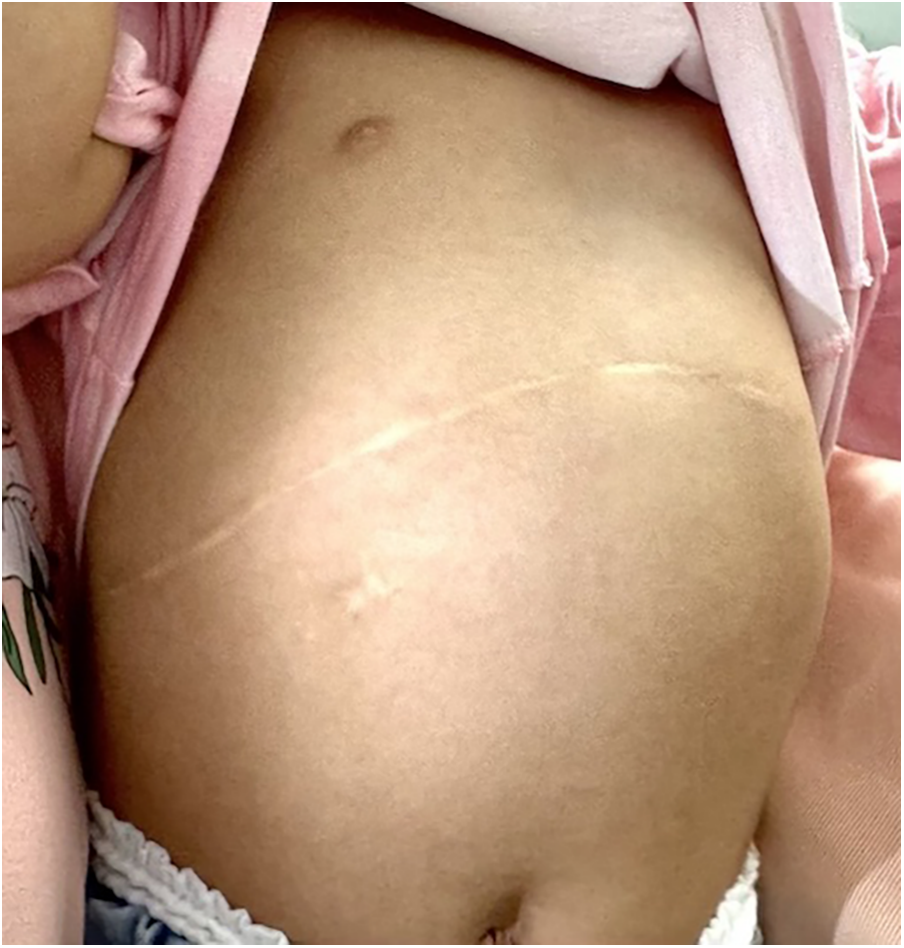
Surgical scars in the control group.

**Figure 4:**
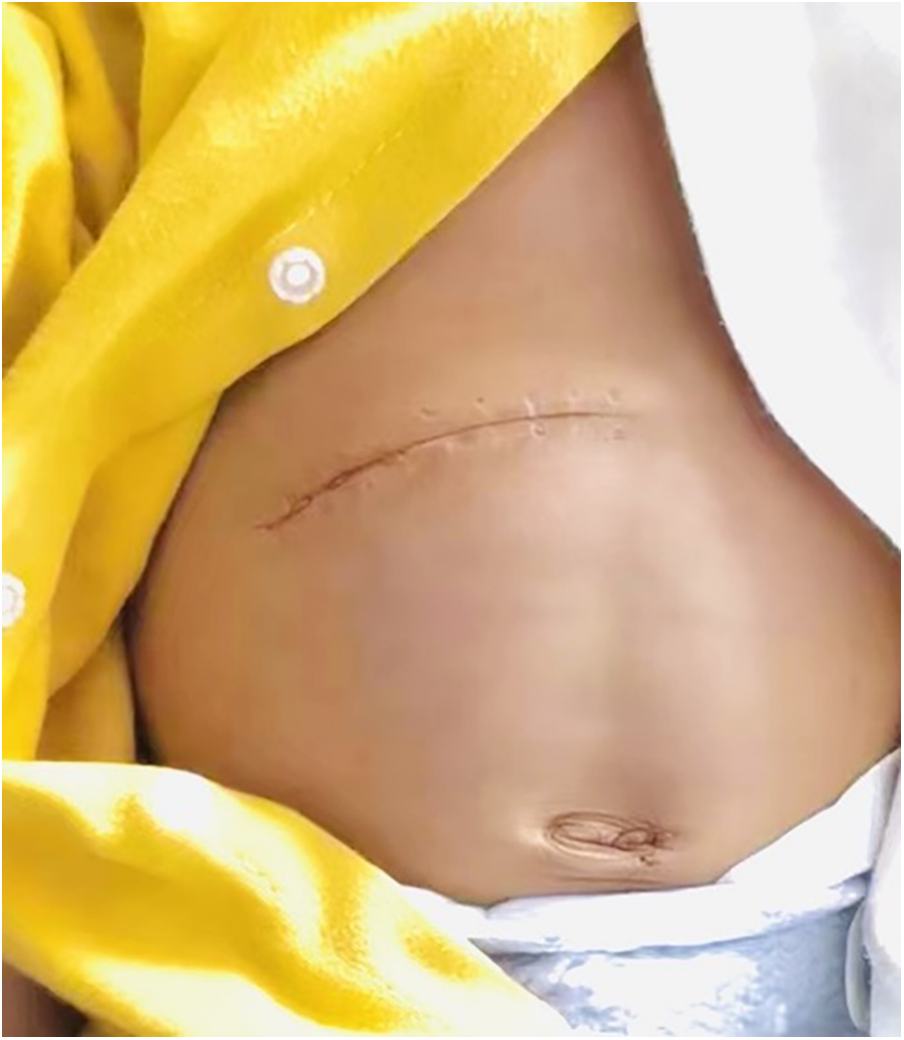
Surgical scars in the experimental group group.

The above advantages are related to the following aspects: (1) Traditional Kasai surgery may result in obstruction of venous return, decreased hepatic blood flow, and decreased cardiac blood flow during the process of lifting the liver out of the abdominal cavity, as evidenced by a significant decrease in intraoperative blood pressure levels. On the other hand, lifting the liver may stimulate the vagus nerve, thereby impacting circulating blood pressure, and postoperative retraction of the liver back into the abdominal cavity may result in ischemia-reperfusion injury (12). In addition, when removing the liver from the abdominal cavity, there is an increase in the area of heat dissipation, which can lead to a more pronounced drop in body temperature (13). Intraoperative hypothermia increases the risk of surgery (14). (2) The experimental group did not require complete freeing of the perihepatic ligament or lifting of the liver out of the abdominal cavity,so only a smaller surgical incision is needed to meet the surgical requirements. The surgical incision is more aesthetically pleasing, which improves the satisfaction of the patient's family (Figures 3, 4). Less invasive procedure in the experimental group,theoretically greatly reduces the occurrence of postoperative adhesions, and to a certain extent, may reduce the operative time and difficulty of possible liver transplantation in the future(3) The postoperative abdominal drainage volume of the children in the control group was significantly higher than that in the experimental group, which may be due to complete freeing of the perihepatic ligament and most of the superficial lymphatics on the surface of the diaphragm flowing back to the thoracic duct through the perihepatic ligament, eventually causing increased peritoneal drainage. Lymph fluid is rich in protein, and the loss of lymphatic fluid leads to a decrease in protein in the plasma, thus reducing the osmotic pressure in plasma colloids and further increasing the peritoneal effusion, which forms a vicious circle (15). (4) The number of postoperative hospital stays in the experimental group was significantly smaller than that in the control group, which may benefit from a more stable surgical anesthesia process mentioned above and less postoperative abdominal drainage, smaller surgical incisions in the experimental group, so that the children recovered more smoothly. The shorter hospitalization time reduces the financial burden and time commitment from the child's family to a certain extent. (5) There was no significant difference in preoperative and postoperative AST, ALT, γ-GGT, ALP between the experimental group and the control group, the probable reason is that even successful surgery cannot shortly reverse the long-term and progressive liver damage in children with biliary atresia. Long-term follow-up and research are still needed to further clarify the difference between the two.

In this study, 21 patients (67.7%) in the control group did not undergo liver transplantation 1 year after surgery, and all of them survived. In the experimental group, 23 patients (74.2%) survived without liver transplantation at 1 year postoperatively. In the control group, 18 patients (58.1%) had no liver transplantation at 2 years postoperatively, and all survived. In the experimental group, 20 patients (64.5%) did not perform liver transplantation 2 years after surgery, and all of them survived. However, the number of children in this study was small, and the long-term prognosis still requires further study.

In summary, for children with type III biliary atresia, it is more advantageous not to lift the liver out of the abdominal cavity during the Kasai surgery, with a safer surgical anesthesia process, a more aesthetic surgical incision, a reduction in the number of postoperative abdominal drains and postoperative hospitalizations, and without decrease in the outcome of the procedure or prolongation of the surgical time.

## Data Availability

The datasets presented in this study can be found in online repositories. The names of the repository/repositories and accession number(s) can be found in the article/Supplementary Material.
